# Crystal structure and Hirshfeld surface analysis of (2*E*)-3-(4-chloro-3-fluoro­phen­yl)-1-(3,4-di­meth­oxy­phen­yl)prop-2-en-1-one

**DOI:** 10.1107/S2056989019007783

**Published:** 2019-06-04

**Authors:** Sevim Türktekin Çelikesir, S. N. Sheshadri, Mehmet Akkurt, C. S. Chidan Kumar, M. K. Veeraiah

**Affiliations:** a Department of Physics, Faculty of Sciences, Erciyes University, 38039 Kayseri, Turkey; b Department of Chemistry, GSSS Institute of Engineering & Technology for Women, Mysuru 570016, Karnataka, India; c Department of Chemistry, Vidya Vikas Institute of Engineering & Technology, Visvesvaraya Technological University, Alanahalli, Mysuru 570028, Karnataka, India; d Department of Chemistry, Sri Siddhartha Institute of Technology, Tumkur 572 105, Karnataka, India

**Keywords:** crystal structure, *E* configuration, 4-chloro-3-fluoro­phenyl ring, 3,4-di­meth­oxy­phenyl ring, disorder, Hirshfeld surface analysis

## Abstract

The mol­ecular structure of the title compound, C_17_H_14_ClFO_3_, consists of a 4-chloro-3-fluoro­phenyl ring and a 3,4-di­meth­oxy­phenyl ring linked *via* a prop-2-en-1-one spacer. The mol­ecule has an *E* configuration about the C=C bond and the carbonyl group is *syn* with respect to the C=C bond. The F and H atoms at the *meta* positions of the 4-chloro-3-fluoro­phenyl ring are disordered over two orientations, with an occupancy ratio of 0.785 (3):0.215 (3). In the crystal, mol­ecules are linked *via* pairs of C—H⋯O inter­actions with an *R* ^2^_2_(14) ring motif, forming inversion dimers. The dimers are linked into a tape structure running along [10] by a C—H⋯π inter­action. The inter­molecular contacts in the crystal were further analysed using Hirshfield surface analysis, which indicates that the most significant contacts are H⋯H (25.0%), followed by C⋯H/H⋯C (20.6%), O⋯H/H⋯O (15.6%), Cl⋯H/H⋯Cl (10.7%), F⋯H/H⋯F (10.4%), F⋯C/C⋯F (7.2%) and C⋯C (3.0%).

## Abstract 2

The mol­ecule has an *E* configuration about the C=C bond and the carbonyl group is *syn* with respect to the C=C bond. In the crystal, the mol­ecules are connected into a tape structure by C—H⋯O and C—H⋯π inter­actions.

## Chemical context   

Chalcones, compounds with a 1,3-di­phenyl­prop-2-en-1-one framework, are considered to be the precursors of flavonoids and isoflavonoids, which are abundant in edible plants. These compounds are coloured *via* the –CO—CH=CH– chromophore and other auxochromes. Chalcones attract significant attention because of their availability of high optical non-linearities arising from the delocalization of π-conjugated electron clouds throughout the chalcone system, which provides a large charge-transfer axis with appropriate substituents on the terminal aromatic rings. π-conjugated systems have been studied extensively for their optoelectronic properties (Shetty *et al.*, 2016 ▸, 2017 ▸) because of the possibility of developing low-cost, large-area and flexible electronic devices. In view of all the above and in a continuation of our previous work on 3,4-dimeth­oxy chalcones (Sheshadri *et al.*, 2018*a* ▸,*b* ▸), we report herein the crystal and mol­ecular structure of the title compound.
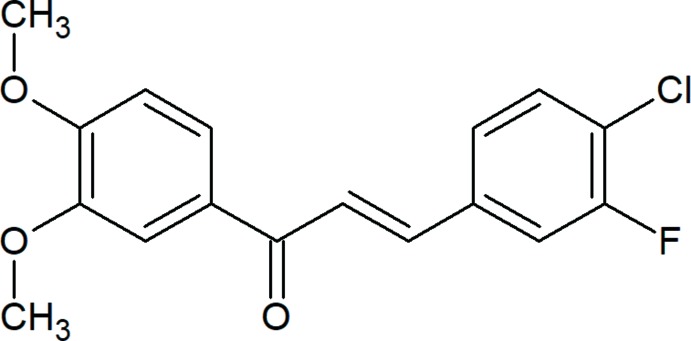


## Structural commentary   

**Figure 1:**
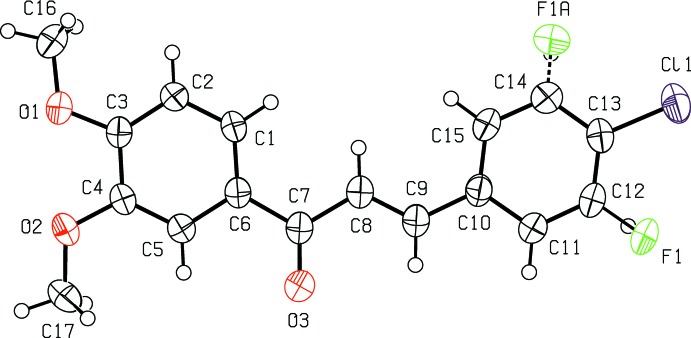
The mol­ecular structure of the title compound, showing the atom labelling and displacement ellipsoids drawn at the 50% probability level.

The title compound (Fig. 1 ▸) is composed of two aromatic rings, 4-chloro-3-fluoro­phenyl and 3,4-di­meth­oxy­phenyl, which are linked by a –CO—CH=CH– enone bridge. The mol­ecule is approximately planar as indicated by the torsion angles C1—C6—C7—O3 = 174.71 (16)°, C1—C6—C7—C8 = −3.8 (2)°, C6—C7—C8—C9 = 178.49 (15)°, O3—C7—C8—C9 = 0.0 (3)°, C8—C9—C10—C11 = 178.22 (17)° and C7—C8—C9—C10 = −179.00 (15)°. The dihedral angle between the 4-chloro-3-fluoro­phenyl and 3,4-di­meth­oxy­phenyl rings is 5.40 (7)°. The H atoms of the central propenone group are *trans* configured. The two meth­oxy groups attached to atoms C3 and C4 are almost coplanar with the benzene ring, with deviations of 0.214 (2) Å for C16 and 0.209 (2) Å for C17. The 4-chloro-3-fluoro­phenyl fragment is disordered over two orientations around the C9—C10 bond axis, with an occupancy ratio of 0.785 (3):0.215 (3).

## Supra­molecular features and Hirshfeld surface analysis   

**Figure 2:**
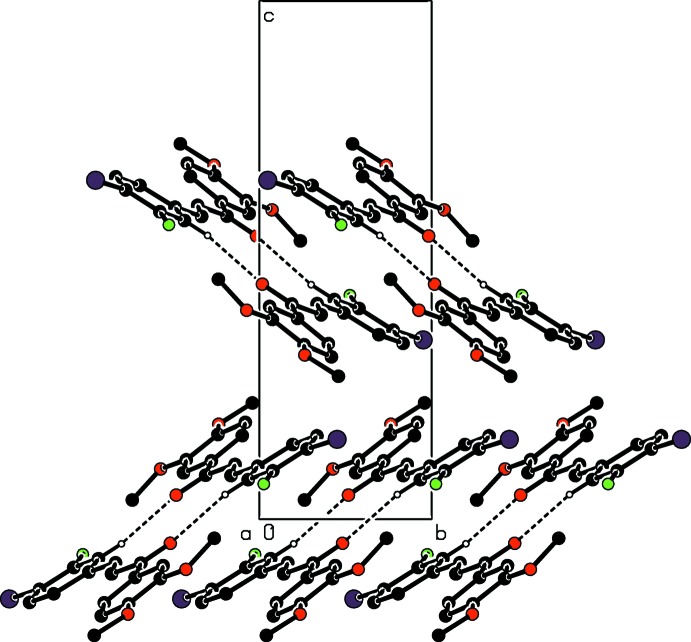
A packing diagram of the title compound viewed along the *a* axis, showing mol­ecular dimers formed by the inter­molecular C—H⋯O hydrogen bonds (dashed lines). The minor disorder component and H atoms not involved in the hydrogen bonds are omitted for clarity.

**Figure 3:**
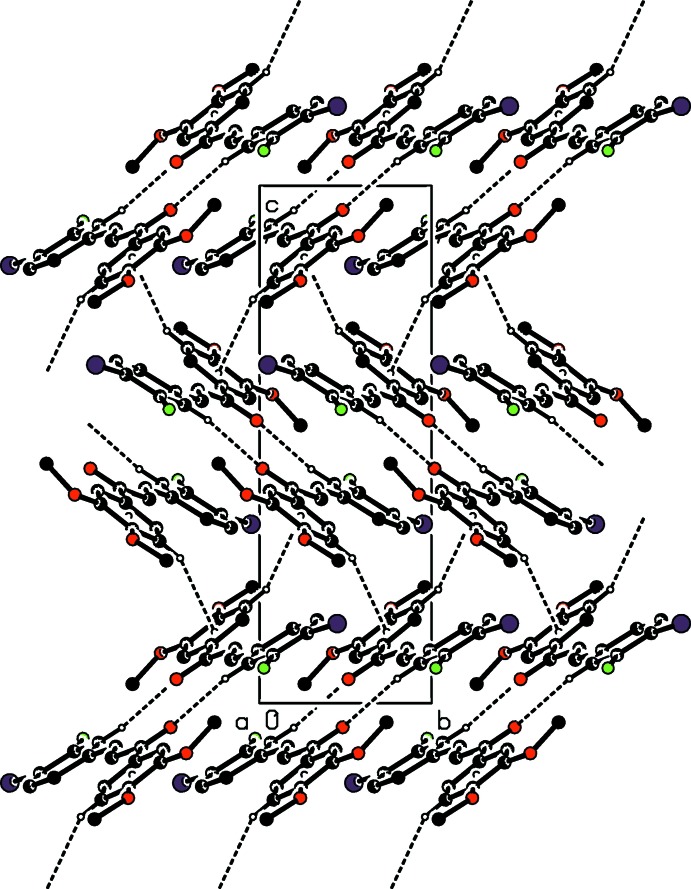
A packing diagram of the title compound viewed along the *a* axis, showing inter­molecular C—H⋯O and C—H⋯π inter­actions (dashed lines). The minor disorder component and H atoms not involved in the hydrogen bonds are omitted for clarity.

In the crystal, the mol­ecules are connected into inversion dimers with an 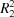
(14) ring motif (Fig. 2 ▸) *via* pairs of C—H⋯O inter­actions (Table 1 ▸). The dimers are further linked by a C—H⋯π inter­action (Table 1 ▸), forming a tape structure along [10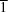] (Fig. 3 ▸).

**Figure 4:**
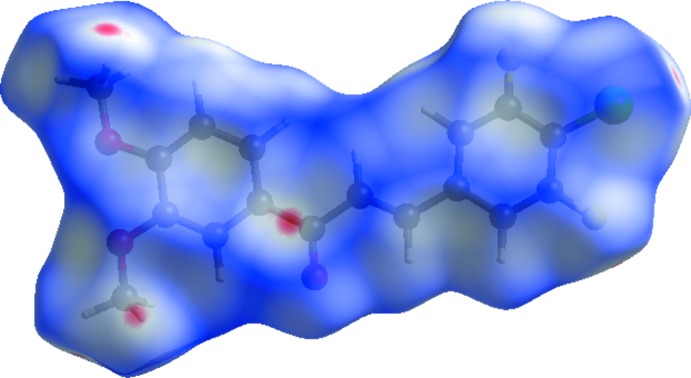
Plot of *d*_norm_ mapped on the Hirshfeld surfaces of the title compound showing the short H⋯O contacts.

**Figure 5:**
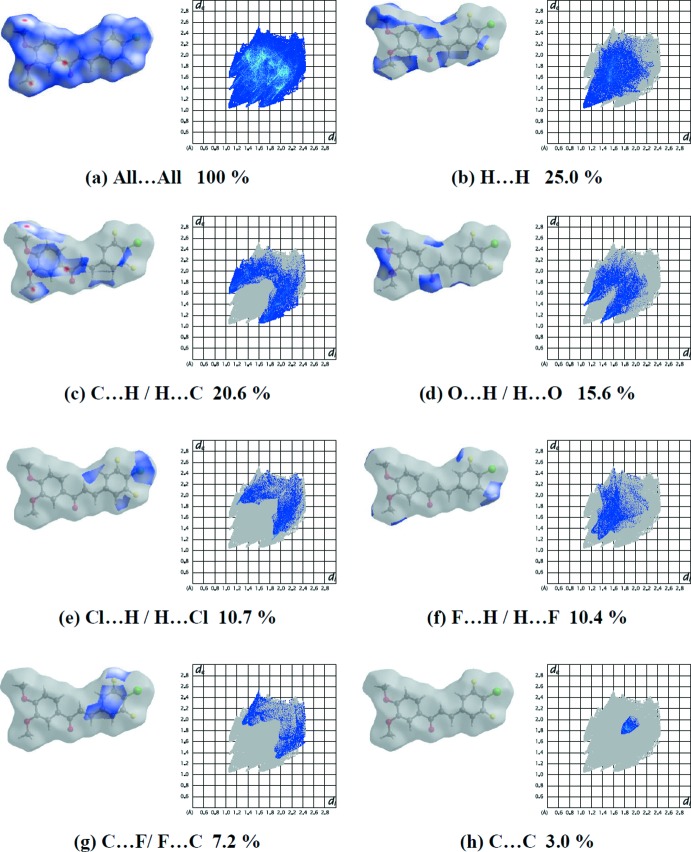
Hirshfeld surface representations and the overall two-dimensional fingerprint plots of the title compound, showing (*a*) all inter­actions, and delineated into (*b*) H⋯H, (*c*) C⋯H/H⋯C, (*d*) O⋯H/H⋯O, (*e*) Cl⋯H/H⋯Cl, (*f*) F⋯H/H⋯F, (*g*) F⋯C/C⋯F and (*h*) C⋯C inter­actions [*d*_e_ and *d*_i_ represent the distances from a point on the Hirshfeld surface to the nearest atoms outside (external) and inside (inter­nal) the surface, respectively].

The Hirshfeld surface and two-dimensional fingerprint plots of the title compound were calculated using *CrystalExplorer17.5* (Turner *et al.*, 2017 ▸). In the Hirshfeld surface plotted over *d*_norm_ (Fig. 4 ▸), the white surfaces indicate contacts with distances equal to the sum of van der Waals radii, and the red and blue colours indicate distances shorter or longer than the van der Waals radii, respectively (Venkatesan *et al.*, 2016 ▸). The overall two-dimensional fingerprint plot and those delineated into H⋯H (25.0%), C⋯H/H⋯C (20.6%), O⋯H/H⋯O (15.6%), Cl⋯H/H⋯Cl (10.7%), F⋯H/H⋯F (10.4%), F⋯C/C⋯F (7.2%) and C⋯C (3.0%) contacts (McKinnon *et al.*, 2007 ▸) are illustrated in Fig. 5 ▸ *a*–*h*, respectively. The small percentage contributions from the other different inter­atomic contacts to the Hirshfeld surfaces are as follows: Cl⋯O/O⋯Cl (2.7%), O⋯C/C⋯O (1.7%), Cl⋯C/C⋯Cl (1.1%), F⋯F (0.9%), Cl⋯F/F⋯Cl (0.7%) and F⋯O/O⋯F (0.2%).

## Database survey   

A search of the Cambridge Structural Database (CSD, version 5.40, update of February 2019; Groom *et al.*, 2016 ▸) using (*E*)-1,3-di­phenyl­prop-2-en-1-one as the main skeleton revealed 3314 hits. Six structures containing the (*E*)-1,3-di­phenyl­prop-2-en-1-one framework with different substituents that are similar to the title compound were found, *viz.* 3-(3-chloro­phen­yl)-1-(3,4-di­meth­oxy­phen­yl)prop-2-en-1-one (VIDVEM; Sheshadri *et al.*, 2018*a* ▸), 3-(3-bromo-4-fluoro­phen­yl)-1-(3,4-di­meth­oxy­phen­yl)prop-2-en-1-one (BIBWOB; Sheshadri *et al.*, 2018*b* ▸), (*E*)-3-(2-bromo­phen­yl)-1-(3,4-di­meth­oxy­phen­yl)prop-2-en-1-one (LAPREB; Li *et al.*, 2012 ▸), (*E*)-1-(3,5-di­fluoro­phen­yl)-3-(2,4-di­meth­oxy­phen­yl)prop-2-en-1-one (KUZFOB; Huang *et al.*, 2010 ▸), (*E*)-1-(3-bromo­phen­yl)-3-(3,4-di­meth­oxy­phen­yl)prop-2-en-1-one (LAQWUX; Escobar *et al.*, 2012 ▸) and 3-(3,4-di­meth­oxy­phen­yl)-1-(4-fluoro­phen­yl)-prop-2-en-1-one(MEGQOF; Butcher *et al.*, 2006 ▸).

For these similar compounds, the dihedral angles between the two terminal benzene rings, which are linked by a –CO—CH=CH– enone bridge are 18.46 (7)° for VIDVEM, 17.91 (17)° for BIBWOB, 9.3 (2) and 19.4 (2)° (two crystallographically independent mol­ecules) for LAPREB, 5.46 (2)° for KUZFOB, 26.59 (9)° for LAQWUX and 47.81 (6) and 50.18 (5)° (two crystallographically independent mol­ecules) for MEGQOF. In the crystals of VIDVEM and BIBWOB, mol­ecules are linked by C—H⋯O hydrogen bonds, forming dimers with 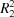
(14) ring motifs, and the dimers are further linked by other C—H⋯O hydrogen contacts, forming two-dimensional supra­molecular structures. In the crystal of LAPREB, mol­ecules are also linked through weak inter­molecular C—H⋯O hydrogen bonds. The crystal structure of KUZFOB is stabilized by inter­molecular C—H⋯F hydrogen bonds.

## Synthesis and crystallization   

The title compound was synthesized as per the procedure reported earlier (Kumar *et al.*, 2013*a* ▸,*b* ▸). 1-(3,4-Di­meth­oxy­phen­yl)ethanone (0.01 mol) and 4-chloro-3-fluoro­benzaldehyde (0.01 mol) were dissolved in 20 ml of methanol. A catalytic amount of NaOH was added to the solution dropwise with vigorous stirring. The reaction mixture was stirred for about 3 h at room temperature. The formed crude products were filtered, washed successively with distilled water and recrystallized from methanol to get the title compound (m.p. 384–388 K).

## Refinement   

Crystal data, data collection and structure refinement details are summarized in Table 2 ▸. The C-bound H atoms were positioned geometrically (C—H = 0.93 or 0.96 Å) and refined using a riding model, with *U*_iso_(H) = 1.2 or 1.5*U*_eq_(C). The 4-chloro-3-fluoro­phenyl fragment was found to be disordered in a difference-Fourier map, and the F and H atoms at the *meta* positions of the benzene ring were treated as disordered over two sites with an occupancy ratio of 0.785 (3):0.215 (3).

## Supplementary Material

Crystal structure: contains datablock(s) I. DOI: 10.1107/S2056989019007783/is5515sup1.cif

Structure factors: contains datablock(s) I. DOI: 10.1107/S2056989019007783/is5515Isup2.hkl

Supporting information file. DOI: 10.1107/S2056989019007783/is5515Isup3.cml

CCDC reference: 1919577

Additional supporting information:  crystallographic information; 3D view; checkCIF report

## Figures and Tables

**Table 1:** Hydrogen-bond geometry (Å, °) *Cg*1 is the centroid of the C1–C6 benzene ring.

*D*—H⋯*A*	*D*—H	H⋯*A*	*D*⋯*A*	*D*—H⋯*A*
C11—H11⋯O3^i^	0.93	2.57	3.426 (2)	152
C2—H2⋯*Cg*1^ii^	0.93	2.81	3.5832 (16)	142

Symmetry codes: (i) 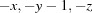; (ii) 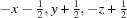.

**Table 2:** Experimental details

Crystal data	
Chemical formula	C_17_H_14_ClFO_3_
*M*_r_	320.73
Crystal system, space group	Monoclinic, *P*2_1_/*n*
Temperature (K)	294
*a*, *b*, *c* (Å)	14.9088 (13), 5.7669 (5), 17.9074 (15)
β (°)	104.491 (2)
*V* (Å^3^)	1490.7 (2)
*Z*	4
Radiation type	Mo *K*α
μ (mm^−1^)	0.28
Crystal size (mm)	0.45 × 0.37 × 0.30

Data collection	
Diffractometer	Bruker APEXII CCD
Absorption correction	Multi-scan (*SADABS*; Sheldrick, 2007 ▸)
*T*_min_, *T*_max_	0.884, 0.921
No. of measured, independent and observed [*I* > 2σ(*I*)] reflections	16268, 4334, 3195
*R*_int_	0.024
(sin θ/λ)_max_ (Å^−1^)	0.704

Refinement	
*R*[*F* ^2^ > 2σ(*F* ^2^)], *wR*(*F* ^2^), *S*	0.047, 0.143, 1.05
No. of reflections	4334
No. of parameters	211
H-atom treatment	H-atom parameters constrained
Δρ_max_, Δρ_min_ (e Å^−3^)	0.33, −0.36

Computer programs: *APEX2* and *SAINT* (Bruker, 2007 ▸), *SHELXS97* (Sheldrick, 2008 ▸), *SHELXL2014* (Sheldrick, 2015 ▸), *ORTEP-3 for Windows* (Farrugia, 2012 ▸) and *PLATON* (Spek, 2009 ▸).

## supplementary crystallographic information

### Crystal data

**Table:**

C_17_H_14_ClFO_3_	*F*(000) = 664
*M**_r_* = 320.73	*D*_x_ = 1.429 Mg m^−^^3^
Monoclinic, *P*2_1_/*n*	Mo *K*α radiation, λ = 0.71073 Å
*a* = 14.9088 (13) Å	Cell parameters from 5099 reflections
*b* = 5.7669 (5) Å	θ = 2.4–29.4°
*c* = 17.9074 (15) Å	µ = 0.28 mm^−^^1^
β = 104.491 (2)°	*T* = 294 K
*V* = 1490.7 (2) Å^3^	Block, colourless
*Z* = 4	0.45 × 0.37 × 0.30 mm

### Data collection

**Table:**

Bruker APEXII CCD diffractometer	3195 reflections with *I* > 2σ(*I*)
φ and ω scans	*R*_int_ = 0.024
Absorption correction: multi-scan (SADABS; Sheldrick, 2007)	θ_max_ = 30.0°, θ_min_ = 1.6°
*T*_min_ = 0.884, *T*_max_ = 0.921	*h* = −19→20
16268 measured reflections	*k* = −8→7
4334 independent reflections	*l* = −25→25

### Refinement

**Table:**

Refinement on *F*^2^	0 restraints
Least-squares matrix: full	Hydrogen site location: inferred from neighbouring sites
*R*[*F*^2^ > 2σ(*F*^2^)] = 0.047	H-atom parameters constrained
*wR*(*F*^2^) = 0.143	*w* = 1/[σ^2^(*F*_o_^2^) + (0.0736*P*)^2^ + 0.2421*P*] where *P* = (*F*_o_^2^ + 2*F*_c_^2^)/3
*S* = 1.05	(Δ/σ)_max_ < 0.001
4334 reflections	Δρ_max_ = 0.33 e Å^−^^3^
211 parameters	Δρ_min_ = −0.36 e Å^−^^3^

### Special details

**Table:**

Geometry. All esds (except the esd in the dihedral angle between two l.s. planes) are estimated using the full covariance matrix. The cell esds are taken into account individually in the estimation of esds in distances, angles and torsion angles; correlations between esds in cell parameters are only used when they are defined by crystal symmetry. An approximate (isotropic) treatment of cell esds is used for estimating esds involving l.s. planes.

### Fractional atomic coordinates and isotropic or equivalent isotropic displacement parameters (Å^2^)

**Table:**

	*x*	*y*	*z*	*U*_iso_*/*U*_eq_	Occ. (<1)
Cl1	0.36793 (3)	0.45137 (9)	0.15399 (3)	0.06876 (17)	
F1	0.34304 (9)	0.0254 (3)	0.06801 (10)	0.0765 (6)	0.785 (3)
F1A	0.1946 (4)	0.5065 (11)	0.2007 (4)	0.094 (2)	0.215 (3)
O1	−0.44876 (7)	−0.2364 (2)	0.18315 (7)	0.0525 (3)	
O2	−0.42868 (8)	−0.5737 (2)	0.09682 (7)	0.0534 (3)	
O3	−0.11186 (9)	−0.4829 (2)	0.04648 (8)	0.0690 (4)	
C1	−0.21547 (10)	−0.0994 (3)	0.16131 (8)	0.0438 (3)	
H1	−0.168526	0.009416	0.177135	0.053*	
C2	−0.29625 (10)	−0.0816 (3)	0.18676 (8)	0.0439 (3)	
H2	−0.302640	0.038463	0.219715	0.053*	
C3	−0.36666 (9)	−0.2405 (2)	0.16343 (8)	0.0388 (3)	
C4	−0.35591 (9)	−0.4244 (2)	0.11467 (7)	0.0392 (3)	
C5	−0.27631 (10)	−0.4408 (2)	0.08964 (8)	0.0412 (3)	
H5	−0.269807	−0.561469	0.056957	0.049*	
C6	−0.20461 (9)	−0.2784 (2)	0.11254 (8)	0.0400 (3)	
C7	−0.11934 (10)	−0.3123 (3)	0.08476 (9)	0.0462 (3)	
C8	−0.04346 (11)	−0.1412 (3)	0.10561 (9)	0.0514 (4)	
H8	−0.050846	−0.012603	0.134851	0.062*	
C9	0.03440 (10)	−0.1648 (3)	0.08409 (9)	0.0468 (3)	
H9	0.038821	−0.294198	0.054228	0.056*	
C10	0.11470 (10)	−0.0096 (3)	0.10207 (8)	0.0437 (3)	
C11	0.19219 (11)	−0.0652 (3)	0.07626 (9)	0.0495 (4)	
H11	0.192714	−0.199152	0.047458	0.059*	
C12	0.26843 (10)	0.0782 (3)	0.09329 (9)	0.0503 (4)	
H12A	0.319949	0.038662	0.075551	0.060*	0.215 (3)
C13	0.27083 (10)	0.2763 (3)	0.13536 (9)	0.0484 (3)	
C14	0.19390 (13)	0.3344 (3)	0.16078 (11)	0.0613 (4)	
H14	0.194093	0.469027	0.189426	0.074*	0.785 (3)
C15	0.11650 (12)	0.1938 (3)	0.14396 (11)	0.0597 (4)	
H15	0.064721	0.235966	0.160943	0.072*	
C16	−0.46843 (13)	−0.0410 (3)	0.22426 (11)	0.0599 (4)	
H16A	−0.531649	−0.047899	0.227596	0.090*	
H16B	−0.458981	0.098166	0.197789	0.090*	
H16C	−0.428007	−0.040654	0.275248	0.090*	
C17	−0.43044 (13)	−0.7381 (3)	0.03698 (10)	0.0576 (4)	
H17A	−0.487558	−0.823211	0.026665	0.086*	
H17B	−0.379347	−0.843355	0.052900	0.086*	
H17C	−0.425610	−0.658282	−0.008901	0.086*	

### Atomic displacement parameters (Å^2^)

**Table:**

	*U*^11^	*U*^22^	*U*^33^	*U*^12^	*U*^13^	*U*^23^
Cl1	0.0483 (3)	0.0636 (3)	0.0962 (4)	−0.01829 (19)	0.0215 (2)	−0.0083 (2)
F1	0.0457 (8)	0.0828 (11)	0.1146 (12)	−0.0100 (6)	0.0454 (8)	−0.0308 (8)
F1A	0.075 (4)	0.081 (4)	0.137 (5)	−0.023 (3)	0.048 (4)	−0.061 (4)
O1	0.0380 (5)	0.0559 (7)	0.0690 (7)	−0.0088 (5)	0.0234 (5)	−0.0118 (5)
O2	0.0445 (6)	0.0533 (7)	0.0650 (6)	−0.0199 (5)	0.0186 (5)	−0.0151 (5)
O3	0.0533 (7)	0.0692 (8)	0.0944 (9)	−0.0154 (6)	0.0372 (7)	−0.0334 (7)
C1	0.0334 (7)	0.0433 (7)	0.0550 (8)	−0.0086 (5)	0.0120 (6)	−0.0064 (6)
C2	0.0380 (7)	0.0425 (7)	0.0519 (7)	−0.0042 (6)	0.0128 (6)	−0.0098 (6)
C3	0.0314 (6)	0.0423 (7)	0.0430 (6)	−0.0021 (5)	0.0101 (5)	0.0017 (5)
C4	0.0337 (6)	0.0398 (7)	0.0429 (6)	−0.0075 (5)	0.0075 (5)	−0.0002 (5)
C5	0.0388 (7)	0.0416 (7)	0.0441 (7)	−0.0053 (5)	0.0120 (6)	−0.0054 (5)
C6	0.0333 (6)	0.0432 (7)	0.0442 (6)	−0.0045 (5)	0.0111 (5)	−0.0013 (5)
C7	0.0371 (7)	0.0517 (8)	0.0523 (7)	−0.0063 (6)	0.0158 (6)	−0.0053 (6)
C8	0.0404 (7)	0.0550 (9)	0.0635 (9)	−0.0096 (7)	0.0215 (7)	−0.0107 (7)
C9	0.0376 (7)	0.0530 (9)	0.0522 (7)	−0.0065 (6)	0.0159 (6)	−0.0045 (6)
C10	0.0359 (7)	0.0507 (8)	0.0471 (7)	−0.0039 (6)	0.0153 (6)	−0.0011 (6)
C11	0.0406 (8)	0.0534 (9)	0.0586 (8)	−0.0035 (6)	0.0197 (6)	−0.0116 (7)
C12	0.0349 (7)	0.0591 (9)	0.0614 (8)	−0.0020 (6)	0.0206 (6)	−0.0032 (7)
C13	0.0387 (7)	0.0510 (8)	0.0569 (8)	−0.0088 (6)	0.0147 (6)	−0.0008 (6)
C14	0.0558 (10)	0.0581 (10)	0.0773 (11)	−0.0098 (8)	0.0305 (9)	−0.0195 (9)
C15	0.0465 (9)	0.0625 (10)	0.0807 (11)	−0.0084 (7)	0.0360 (8)	−0.0177 (8)
C16	0.0514 (9)	0.0636 (11)	0.0738 (11)	−0.0024 (8)	0.0328 (8)	−0.0113 (8)
C17	0.0550 (9)	0.0507 (9)	0.0636 (9)	−0.0141 (7)	0.0082 (7)	−0.0145 (7)

### Geometric parameters (Å, º)

**Table:**

Cl1—C13	1.7278 (15)	C8—H8	0.9300
F1—C12	1.3367 (18)	C9—C10	1.465 (2)
F1A—C14	1.222 (5)	C9—H9	0.9300
O1—C3	1.3565 (16)	C10—C11	1.3851 (19)
O1—C16	1.416 (2)	C10—C15	1.389 (2)
O2—C4	1.3590 (16)	C11—C12	1.376 (2)
O2—C17	1.4259 (19)	C11—H11	0.9300
O3—C7	1.2202 (19)	C12—C13	1.364 (2)
C1—C6	1.3876 (19)	C12—H12A	0.9300
C1—C2	1.3940 (19)	C13—C14	1.377 (2)
C1—H1	0.9300	C14—C15	1.381 (2)
C2—C3	1.3772 (19)	C14—H14	0.9300
C2—H2	0.9300	C15—H15	0.9300
C3—C4	1.4082 (19)	C16—H16A	0.9600
C4—C5	1.3727 (19)	C16—H16B	0.9600
C5—C6	1.4028 (19)	C16—H16C	0.9600
C5—H5	0.9300	C17—H17A	0.9600
C6—C7	1.4893 (19)	C17—H17B	0.9600
C7—C8	1.477 (2)	C17—H17C	0.9600
C8—C9	1.319 (2)		
			
C3—O1—C16	117.83 (12)	C12—C11—C10	119.90 (14)
C4—O2—C17	117.47 (12)	C12—C11—H11	120.1
C6—C1—C2	120.47 (13)	C10—C11—H11	120.1
C6—C1—H1	119.8	F1—C12—C13	117.94 (14)
C2—C1—H1	119.8	F1—C12—C11	119.95 (15)
C3—C2—C1	120.46 (13)	C13—C12—C11	122.11 (14)
C3—C2—H2	119.8	C13—C12—H12A	118.9
C1—C2—H2	119.8	C11—C12—H12A	118.9
O1—C3—C2	125.34 (13)	C12—C13—C14	118.53 (14)
O1—C3—C4	115.13 (12)	C12—C13—Cl1	120.07 (12)
C2—C3—C4	119.53 (12)	C14—C13—Cl1	121.38 (13)
O2—C4—C5	125.75 (13)	F1A—C14—C13	120.4 (3)
O2—C4—C3	114.52 (12)	F1A—C14—C15	119.3 (3)
C5—C4—C3	119.73 (12)	C13—C14—C15	120.31 (16)
C4—C5—C6	121.10 (13)	C13—C14—H14	119.8
C4—C5—H5	119.5	C15—C14—H14	119.8
C6—C5—H5	119.5	C14—C15—C10	121.06 (14)
C1—C6—C5	118.69 (12)	C14—C15—H15	119.5
C1—C6—C7	123.47 (12)	C10—C15—H15	119.5
C5—C6—C7	117.82 (12)	O1—C16—H16A	109.5
O3—C7—C8	120.58 (13)	O1—C16—H16B	109.5
O3—C7—C6	119.93 (13)	H16A—C16—H16B	109.5
C8—C7—C6	119.47 (13)	O1—C16—H16C	109.5
C9—C8—C7	122.13 (15)	H16A—C16—H16C	109.5
C9—C8—H8	118.9	H16B—C16—H16C	109.5
C7—C8—H8	118.9	O2—C17—H17A	109.5
C8—C9—C10	127.13 (15)	O2—C17—H17B	109.5
C8—C9—H9	116.4	H17A—C17—H17B	109.5
C10—C9—H9	116.4	O2—C17—H17C	109.5
C11—C10—C15	118.09 (14)	H17A—C17—H17C	109.5
C11—C10—C9	119.29 (14)	H17B—C17—H17C	109.5
C15—C10—C9	122.62 (13)		
			
C6—C1—C2—C3	−0.4 (2)	O3—C7—C8—C9	0.0 (3)
C16—O1—C3—C2	7.1 (2)	C6—C7—C8—C9	178.49 (15)
C16—O1—C3—C4	−172.50 (14)	C7—C8—C9—C10	−179.00 (15)
C1—C2—C3—O1	−178.37 (14)	C8—C9—C10—C11	178.22 (17)
C1—C2—C3—C4	1.2 (2)	C8—C9—C10—C15	−2.1 (3)
C17—O2—C4—C5	−11.5 (2)	C15—C10—C11—C12	0.9 (3)
C17—O2—C4—C3	168.72 (13)	C9—C10—C11—C12	−179.46 (15)
O1—C3—C4—O2	−1.91 (18)	C10—C11—C12—F1	−179.59 (16)
C2—C3—C4—O2	178.50 (13)	C10—C11—C12—C13	0.0 (3)
O1—C3—C4—C5	178.26 (13)	F1—C12—C13—C14	179.06 (17)
C2—C3—C4—C5	−1.3 (2)	C11—C12—C13—C14	−0.6 (3)
O2—C4—C5—C6	−179.07 (13)	F1—C12—C13—Cl1	0.4 (2)
C3—C4—C5—C6	0.7 (2)	C11—C12—C13—Cl1	−179.23 (14)
C2—C1—C6—C5	−0.2 (2)	C12—C13—C14—F1A	177.6 (5)
C2—C1—C6—C7	−178.27 (14)	Cl1—C13—C14—F1A	−3.8 (5)
C4—C5—C6—C1	0.0 (2)	C12—C13—C14—C15	0.2 (3)
C4—C5—C6—C7	178.21 (13)	Cl1—C13—C14—C15	178.81 (15)
C1—C6—C7—O3	174.71 (16)	F1A—C14—C15—C10	−176.7 (5)
C5—C6—C7—O3	−3.4 (2)	C13—C14—C15—C10	0.8 (3)
C1—C6—C7—C8	−3.8 (2)	C11—C10—C15—C14	−1.3 (3)
C5—C6—C7—C8	178.12 (14)	C9—C10—C15—C14	179.08 (17)

### Hydrogen-bond geometry (Å, º)

*Cg*1 is the centroid of the C1–C6 benzene ring.

**Table:**

*D*—H···*A*	*D*—H	H···*A*	*D*···*A*	*D*—H···*A*
C11—H11···O3^i^	0.93	2.57	3.426 (2)	152
C2—H2···*Cg*1^ii^	0.93	2.81	3.5832 (16)	142

Symmetry codes: (i) −*x*, −*y*−1, −*z*; (ii) −*x*−1/2, *y*+1/2, −*z*+1/2.
